# Ethnic Variation in Left Ventricular Size and Mechanics During Healthy Pregnancy: A Systematic Review of Asian and Western Cohorts

**DOI:** 10.3390/jcm14248745

**Published:** 2025-12-10

**Authors:** Andrea Sonaglioni, Giovanna Margola, Gian Luigi Nicolosi, Stefano Bianchi, Michele Lombardo, Massimo Baravelli

**Affiliations:** 1 Division of Cardiology, IRCCS MultiMedica, 20123 Milan, Italy; michele.lombardo@multimedica.it (M.L.); massimo.baravelli@multimedica.it (M.B.); 2 Division of Gynaecology and Obstetrics, IRCCS MultiMedica, 20123 Milan, Italy; giovanna.margola@studenti.unimi.it (G.M.); stefano.bianchi@unimi.it (S.B.); 3 Division of Cardiology, Policlinico San Giorgio, 33170 Pordenone, Italy; gianluigi.nicolosi@gmail.com

**Keywords:** pregnancy, global longitudinal strain, ethnic differences, cardiac remodeling, maternal cardiovascular adaptation

## Abstract

**Background**: Pregnancy induces substantial cardiovascular remodeling, yet whether maternal cardiac adaptation differs across ethnic groups remains unclear. Body size, ventricular geometry, and thoracoabdominal configuration may modulate key functional indices such as left ventricular ejection fraction (LVEF) and global longitudinal strain (LV-GLS). This systematic review compared echocardiographic characteristics between Asian and Western healthy pregnant women in late gestation and explored physiological mechanisms underlying observed differences. **Methods**: A comprehensive search of PubMed, Scopus, and EMBASE identified studies reporting transthoracic echocardiography in healthy singleton third-trimester pregnancies across Asian and Western populations. Extracted variables included anthropometry, ventricular dimensions and volumes, LVEF, and LV-GLS. Pooled estimates were calculated using inverse-variance weighting, with heterogeneity quantified using the I^2^ statistic. Study quality was assessed with the NIH Case–Control Quality Assessment Tool. Comparative forest plots visualized population differences. **Results:** Twenty studies involving 1431 participants (578 Asian and 853 Western women) met inclusion criteria. Asian women consistently exhibited smaller ventricular chambers, higher LVEF, and more favorable LV-GLS. Importantly, these differences persisted after indexing LV-GLS to BSA, indicating that body-size normalization attenuates—but does not eliminate—population differences in myocardial deformation. Western women demonstrated slightly attenuated GLS despite preserved LVEF, plausibly attributable to larger cardiac size, higher wall stress, greater diaphragmatic elevation, and increased extrinsic thoracic compression. Between-study heterogeneity was substantial (I^2^ > 95%) due to variation in imaging platforms, strain software, and population characteristics. Methodological quality was fair, with frequent lack of sample-size justification and incomplete confounder adjustment. **Conclusions**: Healthy Asian pregnant women display a hyperdynamic systolic phenotype, whereas Western women show a physiologically appropriate, load-related attenuation of LV-GLS with preserved LVEF. These findings highlight the need for ethnicity-associated and anatomy-aware echocardiographic reference values and support incorporating thoracic geometric indices, such as the modified Haller Index, into strain interpretation during pregnancy.

## 1. Introduction

Cardiovascular disease represents the leading cause of maternal mortality worldwide [1,2]. Approximately two-thirds of these deaths are potentially preventable [3], and it is estimated that 21% are related to cardiac/coronary conditions and cardiomyopathy [4]. Pregnancy imposes substantial hemodynamic stress on the cardiovascular system, including marked increases in cardiac output, heart rate, and circulating blood volume, which may unmask or exacerbate latent cardiac dysfunction. A comprehensive assessment of cardiac structure and function in healthy pregnant women is therefore essential to improve our understanding of physiological cardiac remodeling and to identify deviations that characterize maladaptation, as observed in pregnant women with obesity or in pregnancies complicated by gestational hypertension or gestational diabetes mellitus.

Transthoracic echocardiography (TTE) is the most commonly used screening method for evaluating cardiac function and structure in pregnancy due to its safety and availability. It allows clinicians to obtain information about cardiac chamber size, left ventricular (LV) diastolic function, biventricular systolic performance, valvular function, and pulmonary hemodynamics. During the last decade, a limited number of studies have attempted to define normal echocardiographic parameters during pregnancy; however, these investigations were generally based on small sample sizes and heterogeneous time points across gestation, making it difficult to establish general reference ranges [5,6,7].

Population-based datasets of echocardiographic measurements obtained from large samples of healthy non-pregnant individuals without clinical cardiovascular or renal disease, hypertension, or diabetes have highlighted important ethnic differences in left atrial (LA) and LV dimensions and volumes, left ventricular mass index (LVMi), and left ventricular ejection fraction (LVEF) [8]. Notably, the lower reference value of LVEF for Europeans was 6% lower than that of East Asians and 2% lower than that of South Asians. This difference was statistically significant for both sexes. Moreover, the upper reference values for left ventricular end-diastolic diameter (LVEDD), left ventricular end-systolic diameter (LVESD), left ventricular end-diastolic volume (LVEDV), left ventricular end-systolic volume (LVESV), and LA diameter were higher for Europeans than for Asians.

The higher LVEF values observed in Asians compared with Europeans are likely related to the association between higher LVEF and small cardiac size. This association suggests that individuals with smaller cardiac chambers may rely more heavily on contractile reserve to maintain adequate cardiac output [9]. Additionally, it is noteworthy that LVEF may be overestimated in individuals with normal cardiac function and small hearts [10,11].

To date, ethnic differences in normative echocardiographic values have been poorly investigated during pregnancy. Wilkie G. et al. [12] found higher LVEF in Asian women compared with White women, although cardiac geometry did not differ significantly between groups and all echocardiographic parameters remained within normal limits. The authors attributed the higher LVEF in Asians to their markedly smaller sample size (*n* = 22) compared with Whites (*n* = 204). However, this finding raises the possibility that ethnic differences in cardiac mechanics—well established in non-pregnant populations—may persist during pregnancy and deserve more systematic investigation.

During the past two decades, advances in cardiac imaging have led to the development of speckle-tracking echocardiography (STE), a technique that quantifies myocardial deformation and provides sensitive markers of systolic function [13]. The principal STE-derived index of myocardial contractility is left ventricular global longitudinal strain (LV-GLS). An absolute LV-GLS value <20% in the presence of preserved LVEF (≥55%) is considered diagnostic of subclinical myocardial dysfunction [14]. As far as we know, no previous study has specifically evaluated ethnic differences in LV-GLS among healthy pregnant women, despite the potential of strain analysis to detect subtle functional adaptations.

Based on existing literature, we hypothesized that Asian third-trimester healthy pregnant women might have a higher LVEF than Western third-trimester healthy pregnant women, due to the presumed association between higher LVEF and small cardiac size in Asian ethnicity. Similarly, given the close relationship between myocardial deformation and chamber geometry, we anticipated that LV-GLS might also be significantly more pronounced in Asian vs. European healthy pregnant women. Accordingly, the present systematic review was primarily designed to compare traditional and advanced echocardiographic parameters obtained from TTE studies implemented with STE analysis of LV mechanics in Western vs. Asian third-trimester healthy pregnant women.

## 2. Materials and Methods

The methodology of this systematic review adhered to the standards outlined in the PRISMA statement [15] (Supplementary Materials S1). The study protocol was preregistered in the INPLASY database (ID: INPLASY2025110078) on 25 November 2025 (Supplementary Materials S2).

### 2.1. Search Strategy

Two independent reviewers (A.S. and M.B.) carried out a comprehensive literature search in PubMed, Scopus, and EMBASE from database inception through October 2025. The objective was to identify all studies evaluating cardiac morphology and function in healthy third-trimester pregnant women using transthoracic echocardiography, with or without speckle-tracking echocardiography. The search strategy included combinations of the following terms: “pregnancy” or “pregnant women,” combined with “echocardiography,” “transthoracic echocardiography,” “speckle tracking echocardiography,” “strain imaging,” “left ventricular strain,” “global longitudinal strain,” or “LV mechanics,” and filtered using ethnicity descriptors such as “Asian,” “Chinese,” “Japanese,” “Korean,” “South Asian,” “East Asian,” “Caucasian,” “European,” or “Western.” No language or temporal restrictions were applied. In addition to database searching, the reference lists of all included papers and relevant narrative or systematic reviews were examined manually to identify additional eligible studies. All discrepancies between reviewers were resolved through discussion or, when necessary, consultation with a third investigator.

### 2.2. Eligibility Criteria

Studies were considered eligible when they examined healthy third-trimester pregnant women aged between 18 and 45 years, clearly identified as Asian or Western. Eligible designs included prospective or retrospective observational studies with either cross-sectional or longitudinal structure. To be included, studies had to assess cardiac structure or function using transthoracic echocardiography and provide quantitative measurements of at least one echocardiographic parameter such as LV dimensions, volumes, LVEF, diastolic indices, or speckle-tracking–derived indices including LV-GLS, circumferential strain and radial strain. Studies lacking sufficient information to classify participants into either the Western or Asian group were excluded.

Exclusion criteria included studies enrolling pregnant women younger than 18 or older than 45 years, women not examined during the third trimester, or women whose ethnicity did not match the Asian or Western categories being investigated. Studies involving pregnancies complicated by preexisting hypertension, pregestational diabetes mellitus, chronic pulmonary or renal disease, structural heart disease, cardiomyopathy, congenital heart abnormalities, or fetal abnormalities were excluded, as were pregnancies complicated by gestational hypertension, preeclampsia or gestational diabetes mellitus. Studies were also excluded if participants exhibited hemodynamic instability or if echocardiographic images were judged inadequate. Non-eligible publication types included case reports, small case series, editorials, narrative reviews, conference abstracts, and any study relying exclusively on imaging modalities other than echocardiography.

### 2.3. Study Selection and Data Extraction

Two reviewers (A.S. and M.B.) independently screened all retrieved titles and abstracts and subsequently evaluated the full texts of articles meeting initial eligibility criteria. Data extraction was performed using a standardized form that captured study characteristics (including first author, country, year of publication and study design), population characteristics (such as sample size, maternal age, ethnicity, gestational age at echocardiography, parity, body mass index, body surface area, blood pressure and heart rate), and echocardiographic methodology (including equipment manufacturer, STE software vendor, dimensionality of imaging such as 2D or 3D STE, frame rate, and operator experience). Extracted echocardiographic variables included LV dimensions and volumes, LVEF, LVMi and diastolic indices, as well as all available strain parameters including LV-GLS, left ventricular global circumferential strain (LV-GCS), left ventricular global radial strain (LV-GRS), left atrial reservoir strain (LASr) and right ventricular global longitudinal strain (RV-GLS). Summary statistics were collected as reported in each study. When numerical results were available only in graphical format, values were extracted using digital caliper software. Differences in extracted data were resolved through consensus.

### 2.4. Risk of Bias Assessment

The methodological quality of all included studies was assessed independently by two reviewers (A.S. and G.M.) using the National Institutes of Health (NIH) Quality Assessment Tool for Case-Control Studies [16]. Each study was categorized as having good, fair or poor quality in accordance with the NIH scoring criteria. Inter-rater agreement between the two reviewers was quantified using Cohen’s kappa, and any disagreements were settled through discussion and consensus.

### 2.5. Statistical Analysis

The distribution of continuous variables was assessed using the Shapiro–Wilk test, which showed that most clinical, anthropometric, and echocardiographic parameters were non-normally distributed. Accordingly, continuous variables were summarized using medians with interquartile ranges (IQR), whereas categorical variables were expressed as frequencies and percentages. Between-group comparisons for non-normally distributed continuous variables were conducted using the Mann–Whitney U test, and categorical variables were analyzed using the chi-square test or Fisher’s exact test where appropriate.

For echocardiographic parameters reported as study-level means—such as LVEF, LV-GLS, LV-GCS, LV-GRS, left ventricular volumes and dimensions (LVEDD, LVEDV, LVESV), and LVMi—means and standard deviations were extracted directly from the included studies. To facilitate comparability across publications with heterogeneous reporting formats, myocardial strain values (GLS and GCS) were treated as positive magnitudes, reflecting the absolute degree of deformation rather than directional polarity. In addition to absolute strain values, LV-GLS indexed to body surface area (GLS/BSA) was analyzed to assess whether body-size normalization attenuated or preserved ethnic differences in myocardial deformation. Indexed GLS values were incorporated into forest plots and included in pooled statistical comparisons in parallel with unindexed GLS.

Comparisons of pooled mean values between Asian and Western cohorts were performed using Welch’s t-test, which does not assume equal variances and is appropriate for sample size imbalance and heteroscedasticity between groups. This approach allowed consistent computation of *p*-values across all parameters for which mean and standard deviation were available. Pooled means were derived through inverse-variance weighting, and between-study heterogeneity was quantified using the I^2^ statistic.

To visualize differences in key functional parameters, forest plots for LVEF and LV-GLS were generated using Python (matplotlib), displaying study-specific medians with 95% confidence intervals (calculated from reported means, SD, and sample size) stratified by ethnicity. Vertical dashed reference lines indicated the pooled Western and pooled Asian central values to facilitate direct comparison.

All statistical analyses were performed using IBM SPSS Statistics (version 29.0, Armonk, NY, USA) and Python (version 3.11). A two-sided *p*-value < 0.05 was considered statistically significant.

## 3. Results

### 3.1. Study Selection

**Figure 1:**
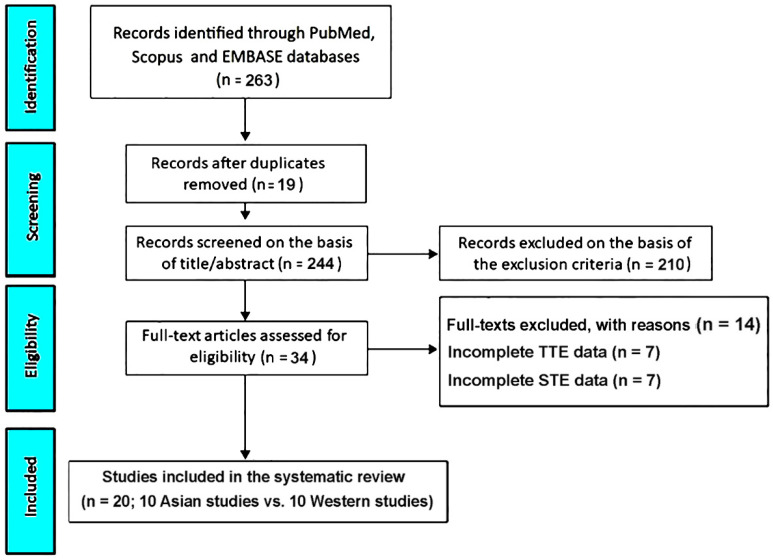
PRISMA flow diagram of study selection for the systematic review.

The database search initially identified 263 records. After removing 19 duplicates (7.2%), 244 unique studies remained. Of these, 210 publications (79.8%) were excluded during title and abstract screening because they did not meet the predefined eligibility criteria. The remaining 34 articles (12.9%) underwent full-text review. During this phase, 7 studies (2.7%) were excluded due to insufficient TTE data, and an additional 7 (2.7%) were excluded for lacking complete STE information. Ultimately, 20 studies—10 from Asian cohorts [17,18,19,20,21,22,23,24,25,26] and 10 from Western cohorts [27,28,29,30,31,32,33,34,35,36]—met all inclusion criteria and were incorporated into the final comparative analysis of healthy pregnant women (Figure 1).

### 3.2. Clinical and Anthropometric Findings

A summary of clinical characteristics and principal findings from Asian and Western studies is presented in Table 1 and Table 2, respectively.

The studies included in this comparative systematic review were published between 2011 and 2025 and were conducted across multiple Asian countries (China, South Korea) and Western regions (Italy, Germany, Sweden, the United Kingdom, Spain, Serbia, Turkey, Norway, and the United States). Together, the Asian cohort comprised 578 healthy third-trimester pregnant women, while the Western cohort included 853 healthy third-trimester pregnant women. All included studies enrolled normotensive, non-diabetic, singleton pregnancies without maternal cardiovascular, renal, or pulmonary disease and uniformly applied TTE as the primary imaging modality. Several studies also incorporated speckle-tracking echocardiography (2D-STE or 3D-STE), introducing some methodological heterogeneity due to differences in ultrasound vendor (GE, Philips, Siemens, TomTec) and strain-analysis software (EchoPAC, QLAB, TomTec, and vendor-specific 3D STE platforms). Gestational age at examination ranged from 28 to 40 weeks, with most imaging performed between 30 and 38 weeks.

Asian studies (Table 1) were largely conducted in China, with one multicenter Korean cohort, and demonstrated consistent methodological rigor across predominantly prospective monocentric designs. These investigations commonly utilized GE-based 2D-STE systems, with some studies employing Siemens or TomTec platforms. A central finding across the Asian literature was a progressive physiological decline in LV-GLS across advancing gestation, documented repeatedly in serial cohorts [18,19,20]. Moreover, Asian studies consistently reported significantly better strain values in healthy pregnancies compared with pregnancies complicated by gestational hypertension, preeclampsia, or gestational diabetes [21,22,23,24,25,26]. Several studies evaluated women in the early third trimester, providing valuable insight into early myocardial adaptation before peak hemodynamic load.

Western studies (Table 2), in contrast, originated primarily from Europe and North America and were also largely prospective monocentric investigations, though with a broader mix of ultrasound and software platforms—GE, Philips, Siemens, and TomTec. Sample sizes ranged from small exploratory cohorts [27] to large population-based studies exceeding 200 participants [31]. Despite this heterogeneity, Western findings were highly consistent: healthy pregnancy was associated with preserved or modestly enhanced LV-GLS compared with pregnancies complicated by preeclampsia, gestational hypertension, gestational diabetes, or anemia. Several Western investigations [28,32] mirrored the Asian literature in documenting physiologic reductions in ventricular mechanics across gestation, reinforcing the cross-regional consistency of normal cardiac remodeling patterns.

Across studies, clear anthropometric differences emerged between Asian and Western healthy pregnant women. Asian women demonstrated significantly lower body mass index (BMI) and smaller body surface area (BSA) compared with Western women, consistent with known ethnic differences in body size. These distinctions were not accompanied by major differences in maternal age or parity distribution. From a hemodynamic perspective, heart rate tended to be slightly higher among Asian participants, whereas systolic and diastolic blood pressures were comparable between groups, confirming overall physiological cardiovascular adaptation in both cohorts. All studies reported participants as clinically healthy, with no evidence of gestational hypertension, preeclampsia, or gestational diabetes mellitus.

### 3.3. Maternal Characteristics, Anthropometrics, and Hemodynamics

Maternal characteristics and demographic profiles differed significantly between Asian and Western healthy third-trimester pregnant women (Table 3).

Although gestational age at echocardiographic assessment was broadly similar across groups, Western women underwent imaging at slightly later gestational ages, a difference that reached statistical significance (*p* < 0.001). More pronounced differences were observed in baseline demographic and anthropometric measures: Western women were significantly older (*p* < 0.001) and exhibited higher BSA and BMI compared with Asian women (both *p* < 0.001). These findings reflect well-established ethnic differences in maternal body size and underscore the importance of indexing cardiac measurements to body habitus in comparative analyses.

Hemodynamic parameters also showed modest but statistically significant group differences. Asian women demonstrated slightly higher systolic and diastolic blood pressures (both *p* < 0.001) as well as higher resting heart rates (*p* < 0.001), consistent with physiologic adaptations to pregnancy that may vary subtly by ethnicity. Conversely, Western women displayed marginally lower stroke volume index (SVi) and cardiac output index (COi), both significantly different (*p* < 0.001), although these differences remained within normal gestational ranges. Together, these demographic, anthropometric, and hemodynamic findings establish a clear physiologic context that underpins the structural and functional cardiac differences observed between the two populations.

### 3.4. Conventional Echocardiographic Findings

Conventional transthoracic echocardiographic parameters demonstrated consistent structural and functional differences between Asian and Western healthy pregnant women in the third trimester (Table 4).

As shown in Table 4, these differences were statistically significant for nearly all chamber size–related indices (all *p* < 0.001). In line with their smaller overall body size, Asian women exhibited significantly smaller LVEDD, as well as lower LVEDV and LVESV, compared with Western women (all *p* < 0.001). These differences were directionally homogeneous across studies, although the magnitude varied depending on the echocardiographic modality (2D vs. 3D) and availability of volumetric reconstructions. LVMi was also significantly lower in Asian women (*p* < 0.001), consistent with smaller cavity dimensions and myocardial mass.

Despite these geometric differences, global systolic function was preserved in both groups, with all values within established pregnancy-specific normal limits. However, pooled analysis demonstrated a significantly higher LVEF in Asian compared with Western women (*p* < 0.001), regardless of whether it was calculated using biplane Simpson’s method or 3D volumetric analysis. This pattern is consistent with the known inverse relationship between ventricular size and LVEF, whereby smaller chambers often exhibit proportionally higher systolic performance.

Diastolic indices revealed only minimal ethnic differences. Although the E/A ratio and E/e′ ratio were statistically different between groups (*p* < 0.001), the absolute differences were clinically small, and both cohorts remained within normal gestational ranges, indicating preserved diastolic filling in late pregnancy. No significant differences were observed for posterior wall thickness (PW) or relative wall thickness (RWT), both *p* > 0.99, highlighting the similarity in concentric geometric remodeling across groups. Right ventricular systolic indices (e.g., TAPSE) were reported only in Western studies, preventing direct comparison, though values were uniformly normal.

Taken together, these findings confirm the presence of ethnicity-related differences in LV size and mass, while systolic and diastolic function remain normal in both groups. Asian women tend to display smaller ventricular chambers paired with slightly enhanced global systolic performance, as reflected by their higher LVEF.

### 3.5. Myocardial Strain Parameters

Myocardial deformation analysis using speckle-tracking echocardiography further highlighted meaningful ethnic differences in left ventricular mechanics (Table 5).

As shown in Table 5, the pooled magnitude of LV-GLS was higher in Asian women than in Western women, indicating better preservation of longitudinal systolic deformation. This directional difference was consistent across imaging modalities (2D-STE and 3D-STE), ultrasound vendors, and strain-analysis software, suggesting a robust physiological pattern rather than a methodological artifact.

Given the substantial methodological heterogeneity across studies—including differences in equipment, software packages, frame rates, and acquisition protocols—these results should be interpreted primarily in a descriptive framework. Accordingly, the pooled values reported here are intended as summary indicators rather than formal meta-analytic estimates. Forest plots, presented in a subsequent section, are used solely to visualize the wide inter-study variability and to illustrate the overall directionality of findings.

The enhanced LV-GLS observed in Asian women occurred despite their smaller ventricular size and similar blood pressure profiles, suggesting a more favorable systolic deformation pattern. Other strain indices displayed more heterogeneous results: global circumferential strain and global radial strain did not significantly differ between groups (*p* = 0.772 and *p* = 0.078, respectively), although both remained within normal gestational ranges. Left atrial reservoir strain (LASr), available in a subset of studies, was significantly higher in Asian women (*p* < 0.001), suggesting more favorable atrial compliance during late gestation.

Overall, myocardial strain data reveal a consistent functional phenotype in Asian healthy pregnant women: superior longitudinal deformation (LV-GLS), complemented by higher LVEF, supports the interpretation that smaller cardiac chambers may display enhanced contractile efficiency during late pregnancy.

### 3.6. Comparison of Left Ventricular Ejection Fraction Between Asian and Western Pregnant Women

**Figure 2:**
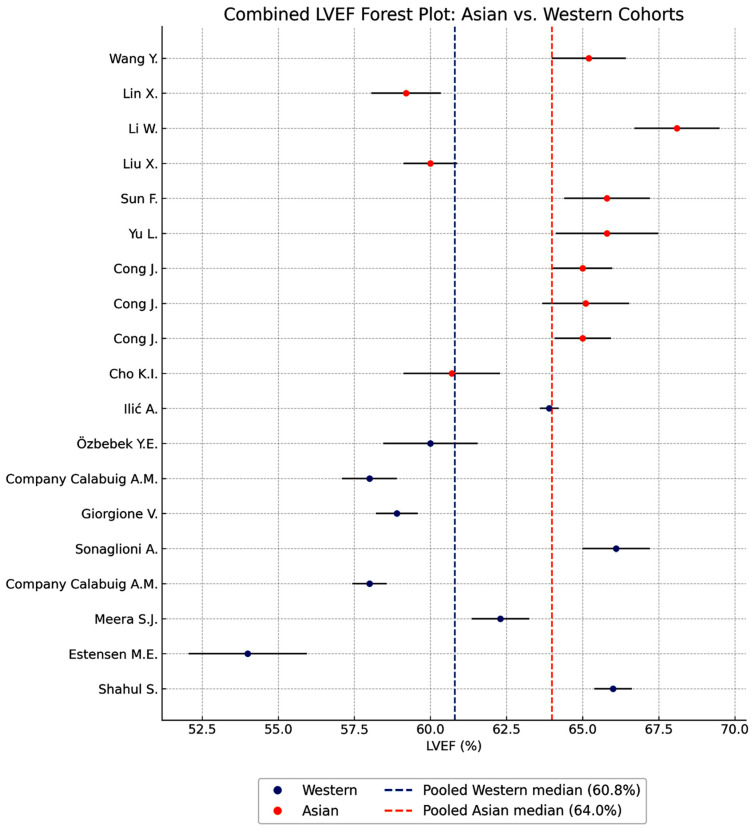
Forest plot comparing left ventricular ejection fraction (LVEF) between Asian (orange) and Western (blue) healthy pregnant women. Grey horizontal lines represent the 95% confidence intervals for each study. The dashed blue and dashed orange vertical lines indicate the pooled Western and Asian median LVEF values (60.8% and 64.0%, respectively).

The combined forest plot for LVEF demonstrates a clear separation between Asian and Western healthy third-trimester pregnant women (Figure 2).

Across individual studies, Asian cohorts consistently demonstrated higher ejection fraction values—generally between ~62% and 68%—while Western cohorts displayed a broader and overall lower distribution, ranging from ~52% to 63%. This pattern persists despite the substantial heterogeneity observed within both groups (I^2^ = 95.86% for Asian studies; 98.96% for Western studies), indicating considerable between-study variability but a stable direction of effect.

The pooled estimates further reinforce this distinction: the pooled Asian LVEF exceeds the pooled Western LVEF, as illustrated by the vertical dashed lines marking each group’s summary value. Nearly all Asian study means lie to the right of the Western pooled reference line, indicating superior systolic performance. Conversely, multiple Western studies cluster to the left, reflecting comparatively reduced contractile function.

Overall, the figure reveals a consistent ethnic pattern, with Asian pregnant women exhibiting higher LVEF in late gestation—a finding likely explained by more efficient systolic mechanics within smaller left ventricular cavities.

### 3.7. Comparison of LV Global Longitudinal Strain Between Asian and Western Pregnant Women

**Figure 3:**
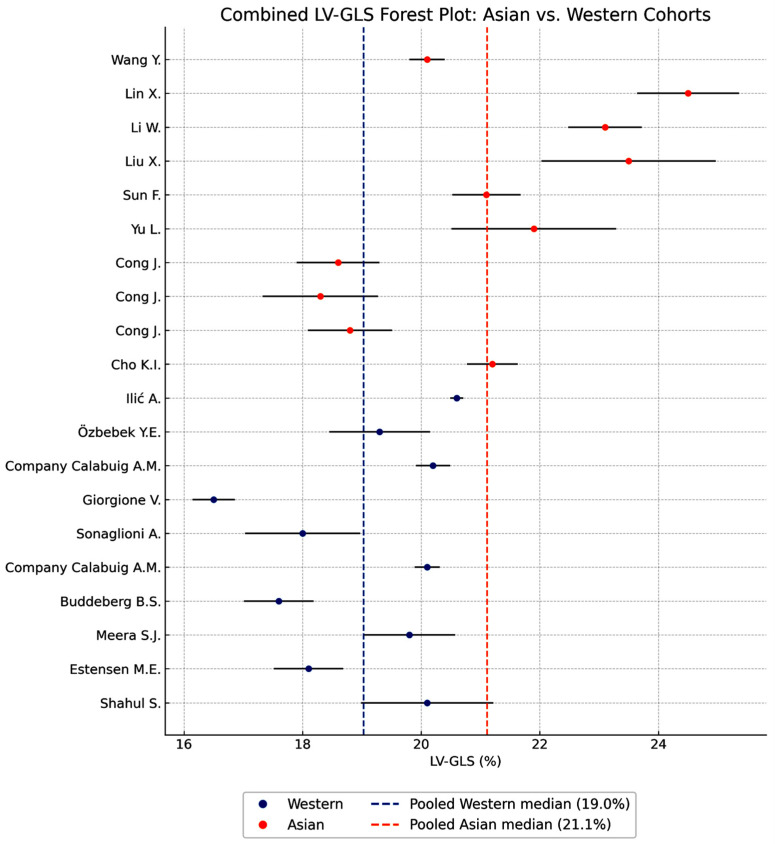
Forest plot comparing LV-GLS between Asian (orange) and Western (blue) healthy pregnant women. Grey horizontal lines represent the 95% confidence intervals for each study. The dashed blue and dashed orange vertical lines indicate the pooled Western and Asian median LV-GLS values (19.0% and 21.1%, respectively).

The combined forest plot for LV-GLS similarly reveals a pronounced distinction between Asian and Western women in late pregnancy (Figure 3).

Asian cohorts consistently exhibit higher absolute GLS values (typically 20% to >24%), indicative of stronger longitudinal myocardial deformation, whereas Western cohorts generally fall within a lower range (~17% to 20%). As observed for LVEF, heterogeneity within both ethnic groups is substantial (I^2^ = 96.51% for Asian studies; 98.52% for Western studies), reflecting considerable between-study variation but a stable and reproducible directional difference.

The pooled estimates further emphasize this pattern: the pooled Asian GLS is clearly higher than the pooled Western GLS, and the vertical dashed line marking the Western pooled value lies to the left of almost all Asian study means. This visual separation is reinforced by the clustered distribution of data points, with Western studies concentrated around lower GLS values and Asian studies forming a distinct cluster to the right, consistent with enhanced myocardial deformation.

**Figure 4:**
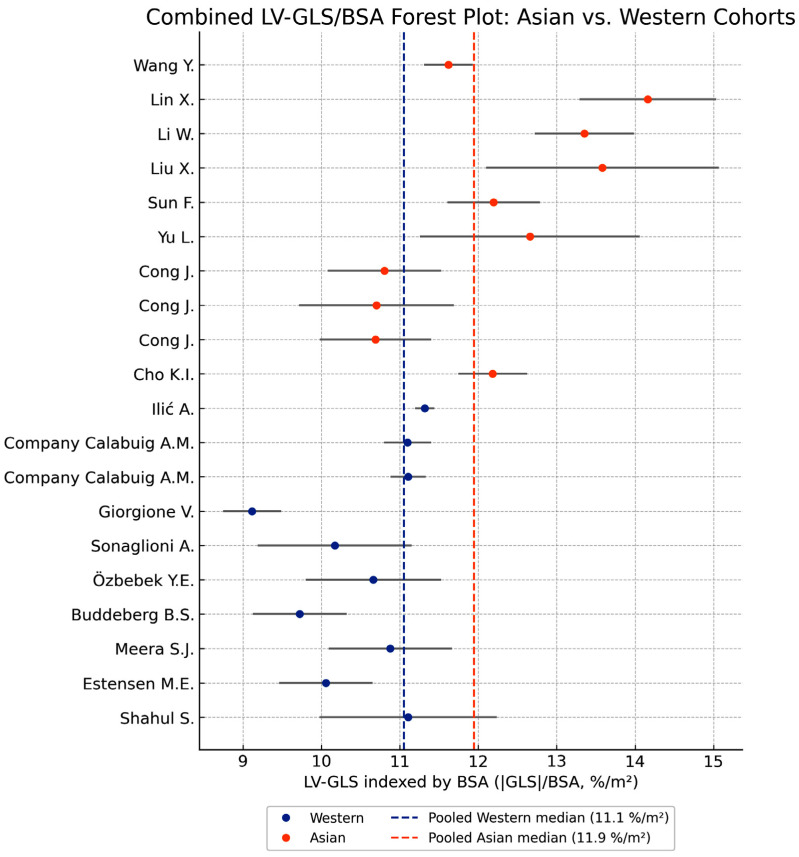
Forest plot comparing left ventricular global longitudinal strain indexed to body surface area (LV-GLS/BSA) between Asian (orange) and Western (blue) healthy pregnant women. Grey horizontal lines represent 95% confidence intervals. The dashed blue and dashed orange vertical lines indicate the pooled Western and Asian median values (11.1%/m^2^ and 11.9%/m^2^, respectively).

A complementary analysis indexing LV-GLS to BSA confirmed the persistence of the ethnic difference, with Asian cohorts maintaining significantly higher strain values than Western cohorts. This is illustrated in Figure 4, which mirrors the pattern observed in Figure 3 but accounts for body size adjustments.

Taken together, these findings demonstrate a robust and consistent ethnic effect, whereby Asian pregnant women exhibit superior LV-GLS in the third trimester. This may reflect more efficient longitudinal systolic mechanics associated with smaller ventricular chamber dimensions.

### 3.8. Publication Bias Assessment

Inter-rater agreement for the methodological quality assessment was substantial, with reviewers providing consistent judgments across all items of the NIH Quality Assessment Tool for Case–Control Studies. As summarized in the Supplementary Materials S3 [17,18,19,20,21,22,23,24,25,26] and S4 [27,28,29,30,31,32,33,34,35,36], the overall methodological quality of the included studies was predominantly fair, and no investigation achieved a “good” rating. Across both groups of studies, the research question, population definition, and case/control criteria were generally well described, and echocardiographic acquisition protocols were implemented consistently.

Several recurrent methodological limitations were identified. Notably, none of the studies reported a sample size justification and no study adequately measured or adjusted for potential confounding variables. Additionally, most investigations did not specify whether the selection of participants was random when fewer than 100% of eligible cases or controls were included. While these limitations reduce internal validity, they do not introduce evidence of systematic directional bias, and the overall pattern of findings remains robust.

## 4. Discussion

### 4.1. Summary of Findings

This comparative systematic review provides the most comprehensive synthesis to date of ethnic variation in maternal cardiac adaptation during late pregnancy, integrating echocardiographic data from more than 2800 healthy Asian and Western third-trimester women. Despite heterogeneity in imaging platforms and analytical methods, a coherent and physiologically plausible pattern of cardiovascular differences emerged across cohorts.

A central observation is that maternal cardiac phenotype in late gestation differs systematically between Asian and Western women at multiple hierarchical levels—demographic, structural, and functional—yet these differences align in a physiologically consistent manner. Asian women, who were characteristically smaller in body size and younger on average, also demonstrated subtly different hemodynamic conditions, including higher heart rates and slightly higher, though still normotensive, systemic pressures. Although these differences were small in absolute magnitude, they help define a distinct physiological milieu that appears to interact with cardiac geometry and function.

Within this anthropometric and hemodynamic context, Asian women consistently exhibited smaller left ventricular dimensions, volumes, and mass, forming a compact cardiac geometry that was reproducible across imaging modalities. Importantly, these structural differences did not translate into impaired performance; instead, they were accompanied by enhanced global systolic indices, including higher ejection fraction and more favorable longitudinal strain. This integrated structural–functional pattern suggests that cardiac efficiency during late pregnancy may scale with body size, with smaller ventricles generating proportionally greater deformation and systolic output relative to their chamber volume. This concept also helps reconcile the seemingly paradoxical observation that Asian women displayed slightly higher brachial systolic and diastolic pressures: when considered within a markedly smaller ventricular radius, these pressures likely produce lower effective end-systolic wall stress, in accordance with Laplace’s law [37], thereby supporting the observed hyperdynamic functional profile.

Strain analysis further reinforced this physiologic interpretation. The markedly higher LV-GLS values observed in Asian women point to a myocardial tissue–level contractile behavior that parallels their smaller ventricular cavities. This relationship between deformation and geometry is consistent with known principles of myocardial mechanics: compact ventricles often demonstrate higher curvature and fiber shortening efficiency, translating into augmented longitudinal strain [38]. The fact that these differences persisted across vendor platforms, strain software, and across both 2D and 3D speckle-tracking techniques suggests that the observed effects are not methodological artefacts, but reflect true physiological variation.

Moreover, the possibility that peripheral brachial pressures do not accurately reflect true central load must be acknowledged; ethnic differences in arterial stiffness, wave-reflection timing, and peripheral resistance—well described in non-pregnant populations [39]—may further contribute to a lower central effective afterload in Asian women despite slightly higher peripheral BP values. This refinement is now incorporated into our mechanistic interpretation as a hypothesis-generating extension requiring future dedicated vascular studies. Notably, the separation between Asian and Western cohorts was statistically significant (Welch’s *p* = 0.0025), confirming that the difference in LV-GLS is unlikely to be attributable to chance alone. Furthermore, indexing LV-GLS to body surface area did not eliminate this effect: Asian women continued to exhibit significantly higher BSA-normalized strain values, with pooled values of 11.9%/m^2^ versus 11.1%/m^2^ in Western cohorts. The persistence of this separation in the BSA-indexed forest plot indicates that the observed differences cannot be attributed solely to simple allometric scaling. While body size undoubtedly contributes to the functional phenotype, the residual difference after normalization suggests additional influences—potentially including thoracoabdominal configuration, myocardial fiber orientation, autonomic tone, or vascular mechanics—beyond scaling alone.

Importantly, the differences in cardiac performance were not explained by disparities in comorbidities—both groups comprised normotensive, non-diabetic low-risk pregnancies—nor by divergent loading conditions, as diastolic filling patterns remained within normal limits and broadly similar between ethnicities. Rather, the findings point toward population-associated variation in cardiac adaptation to pregnancy, potentially influenced by a combination of body size scaling, myocardial geometric properties, vascular reactivity, and genetically mediated determinants of cardiac structure. However, these mechanistic inferences remain provisional, given the cross-sectional and study-level nature of available data; our review cannot disentangle causal pathways nor quantify the relative contributions of scaling versus intrinsic physiological differences.

Across the included literature, methodological limitations—most commonly absence of sample size justification and incomplete adjustment for confounders—were present but did not undermine the consistent directional pattern of results. The reproducibility of findings across geographically diverse cohorts, imaging platforms, and analytic methodologies strengthens confidence that these differences represent authentic physiological variation rather than sampling bias or measurement inconsistency.

Overall, this review identifies a distinct and reproducible cardiovascular phenotype in Asian healthy pregnant women, characterized by smaller ventricular size paired with proportionally enhanced systolic deformation and output. The convergence of absolute and BSA-indexed LV-GLS findings reinforces the physiological relevance of this pattern while highlighting the need for future studies that incorporate more refined allometric modeling, individual-level vascular measurements, and standardized imaging protocols. These findings underscore the need for population-specific reference ranges when assessing maternal cardiac function in late gestation.

### 4.2. Pathophysiological Mechanisms Underpinning Subclinical LV-GLS Impairment in Western Healthy Pregnant Women

The modest reduction in LV-GLS observed among healthy Western pregnant women appears to reflect a multifactorial interaction between intrinsic myocardial adaptation, gestational loading conditions, and population-specific anatomical and biomechanical features. Across Western cohorts, LV-GLS values consistently clustered toward the lower limit of normal despite preserved ejection fraction and absence of structural heart disease. This pattern strongly suggests that the attenuated longitudinal strain reflects physiological remodeling rather than early myocardial dysfunction.

Several hemodynamic and structural characteristics likely contribute. Western women exhibited larger left ventricular chambers, higher stroke volumes, and more pronounced blood volume expansion—factors that elevate preload and alter myocardial fiber stress distribution. The substantial increases in circulating volume and cardiac output during pregnancy redirect wall stress toward mid-wall and circumferential fibers, thereby moderating longitudinal fiber shortening. Moreover, the greater end-diastolic and end-systolic volumes observed in Western cohorts may heighten end-systolic wall stress, placing subtle mechanical constraints on subendocardial longitudinal fibers.

These mechanistic insights are consistent with longitudinal pregnancy studies showing that progressive chamber enlargement and increased stroke work can reduce longitudinal deformation while maintaining stable LVEF [5,6,7]. Thus, the slight GLS reduction in Western women is best understood as a balanced adaptive response to increased loading conditions.

However, the more pronounced GLS difference between Western and Asian women indicates that purely hemodynamic factors do not fully explain the discrepancy. Western women typically have higher BMI and larger thoracoabdominal dimensions, which influence diaphragmatic movement and cardiac position. According to the “mechanical theory” proposed by our group, reductions in longitudinal strain arise partly from extrinsic thoracic and abdominal compression in late gestation [32]. A narrower antero–posterior thoracic diameter, reflected by a higher modified Haller Index (MHI) [40], was strongly associated with reduced strain values, including a robust correlation between GLS and chest narrowing (r = –0.87). These mechanical constraints likely intensify during late gestation as the gravid uterus elevates the diaphragm, abdominal viscera are displaced upward, and the available anterior–posterior thoracic space diminishes.

Western women—characterized by greater adiposity, larger uterine volume, and more substantial diaphragmatic elevation—may therefore experience greater external mechanical load on the ventricles. This can transiently constrain longitudinal deformation despite preserved intrinsic contractile function. Additionally, larger left ventricular volumes tend to produce a more globular ventricular shape. This geometric change alters the incidence of the ultrasound beam, reducing its parallel alignment with the increasingly curvilinear LV walls and the longitudinal direction required for GLS assessment. As a result, GLS values may appear artificially reduced. Importantly, complete postpartum normalization of strain values supports the interpretation that these changes are reversible and mechanistically extrinsic, rather than indicative of cardiomyopathy.

In summary, differences in loading conditions, wall stress, and thoracoabdominal configuration likely act synergistically to explain the slightly lower LV-GLS observed in Western healthy pregnant women despite maintained LVEF and cardiovascular health.

### 4.3. Hypercontractile Systolic Function and Enhanced LV-GLS in Asian Healthy Pregnant Women

The consistently higher LVEF and LV-GLS absolute values documented in Asian healthy pregnant women reflect a coordinated interplay of anatomical, anthropometric, and mechanical factors favoring more efficient systolic performance. Asian women in all included cohorts displayed smaller cardiac chambers, mirroring longstanding ethnic differences in body habitus such as lower BMI and reduced BSA. Smaller ventricles operate on a steeper portion of the end-systolic pressure–volume relationship, enabling greater fiber shortening for any given load [41]. During pregnancy, even modest increases in preload may therefore translate into disproportionally enhanced contractile responses, supporting more favorable longitudinal mechanics.

Ventricular geometry further contributes to this hypercontractile phenotype. A smaller cavity with relatively thicker walls results in lower absolute wall stress, particularly at end-systole. Because longitudinal fibers are highly sensitive to increases in wall stress, the lower-stress environment in smaller ventricles permits more efficient fiber shortening [42]. This protective geometry becomes particularly relevant in late gestation when stroke volume and systolic pressure rise. In contrast, the larger ventricular dimensions typical of Western women amplify wall stress and modestly blunt longitudinal deformation.

Ethnic variation in arterial stiffness, wave-reflection timing, and peripheral resistance—hemodynamic factors capable of modulating central systolic load independently of brachial pressure—may further contribute to lower effective afterload in Asian women despite similar or slightly higher arm-cuff measurements. Although the primary studies did not report pulse-wave velocity or augmentation index, these factors may help reconcile the blood pressure paradox and represent promising avenues for refining the hemodynamic model in future research.

Hemodynamic differences may also reinforce this advantageous contractile pattern. Asian cohorts demonstrated slightly higher heart rates and marginally lower indexed stroke volume, combined with higher cardiac index, suggesting a more dynamic circulatory state that may facilitate enhanced longitudinal fiber shortening. Ethnic differences in autonomic tone and vascular compliance described in non-pregnant populations may persist during pregnancy, contributing to this functional profile.

Additionally, thoracoabdominal geometry may impose fewer mechanical constraints on cardiac motion in Asian pregnant women. A smaller abdominal girth and less diaphragmatic elevation likely reduce extrinsic compression of the heart during late gestation, allowing more complete longitudinal fiber excursion. This contrasts with Western pregnant women, who typically experience greater diaphragmatic elevation and anterior cardiac displacement. Moreover, the naturally more bullet-shaped configuration of a smaller LV may further optimize ultrasound beam incidence in Asian pregnant women, maintaining better alignment with the longitudinal orientation of the LV wall and the trajectory of speckle tracking used for GLS assessment. As a result, GLS measurements in this population may be rendered more accurate and physiologically representative.

An important question is whether these differences merely represent consequences of allometric scaling rather than intrinsic variation in myocardial performance. Smaller ventricles are expected to exhibit greater fractional shortening and higher deformation magnitudes due to geometric scaling principles. However, the persistence of significantly higher LV-GLS values in Asian women even after indexing strain to BSA indicates that body-size normalization attenuates but does not abolish the observed difference, suggesting that myocardial deformation cannot be explained by scaling alone. The BSA-indexed forest plot explicitly demonstrates that a distinct separation between populations remains after normalization, arguing against simple geometric scaling as the sole determinant. Nevertheless, more granular indexing approaches—including height-based indices, lean body mass, and allometric exponents—may reduce residual differences further and should be explored when individual-level data become available.

Collectively, these anatomical, biomechanical, and hemodynamic features form a coherent physiological explanation: healthy Asian pregnant women maintain a structural and loading environment that optimizes myocardial deformation, resulting in a reproducible pattern of higher LVEF and greater GLS magnitude. This pattern appears to arise from the combined—and variably overlapping—effects of compact ventricular geometry, more favorable wall-stress mechanics, ethnicity-associated vascular and autonomic characteristics, and—but not reducible to—body-size scaling. Future studies integrating harmonized echocardiographic protocols with central hemodynamic assessment and allometric modeling will be essential to quantify the relative contribution of each component.

### 4.4. Implications for Clinical Practice

This comparative analysis provides several important insights for the interpretation of echocardiographic findings in pregnancy.

First, the clear ethnic differences in ventricular size, LVEF, and LV-GLS underscore the need to consider population-specific normative ranges when evaluating cardiac function during late gestation [43]. Applying uniform thresholds across diverse populations risks misclassification, especially given that Asian women naturally demonstrate greater GLS and higher LVEF compared with Western women. Clinicians should therefore interpret borderline GLS or LVEF values in the context of ethnic background to avoid overestimating subclinical dysfunction. The persistence of ethnic differences even after BSA-indexing further reinforces that conventional body-size normalization alone may not fully capture physiologic variation across populations, and that ethnicity-associated geometric and vascular factors may meaningfully shift what is “normal” for LV deformation. Importantly, this pattern should not be framed as pathologic “hypercontractility” in Asian women; rather, it represents an efficient physiological state associated with smaller cardiac size, whereas the Western phenotype reflects a normal, load-related attenuation of longitudinal deformation.

Second, the influence of thoracoabdominal geometry and loading conditions highlights the importance of an individualized, anatomy-aware approach to echocardiographic assessment. In Western women—who generally exhibit greater chamber size, elevated wall stress, and more significant diaphragmatic elevation—minor reductions in GLS may reflect reversible mechanical constraints and technical issues, rather than early myocardial impairment. Conversely, a similar reduction in an Asian woman, whose baseline strain is typically higher, may be more clinically meaningful. These findings suggest that mechanical determinants of effective afterload—including ventricular radius, wall thickness, and thoracoabdominal configuration—should be considered alongside brachial blood pressure, which does not reliably reflect central systolic load and may obscure ethnicity-related differences in arterial stiffness or wave-reflection dynamics. Emerging evidence supports the integration of simple structural markers, such as the MHI, into routine assessment to contextualize strain patterns based on thoracic geometry [44]. However, no included primary study reported MHI, thoracic diameters, diaphragmatic position, or ribcage geometry; consequently, the mechanistic interpretation involving thoracic constraint remains hypothesis-generating and based on indirect evidence from differences in chamber size, BSA, and established biomechanical principles rather than direct measurements. This limitation underscores the need for future prospective studies that systematically collect thoracic geometry data. Such individualized interpretation helps avoid unnecessary concern when Asian patients present with high-normal strain values, or when Western patients demonstrate low-normal values that remain physiologically appropriate for their anatomical context.

Finally, as advanced imaging techniques such as speckle-tracking echocardiography become more integral to pregnancy care, these findings support efforts to develop updated, ethnicity-tailored reference intervals for key systolic indices. Incorporating thoracic geometric parameters into strain interpretation may further enhance diagnostic accuracy and improve risk stratification for conditions such as hypertensive disorders of pregnancy and peripartum cardiomyopathy. Future work should also explore whether more granular allometric models—based on height, lean mass, or population-specific scaling exponents—provide superior discrimination compared with traditional BSA-based indexing, thereby improving cross-ethnic comparability of cardiac measurements. A pragmatic initial step may involve parallel development of ethnicity-specific reference ranges and anatomy-based strata (e.g., BSA or MHI quantiles), allowing clinicians to adopt the most feasible approach within their practice while longer-term, geometry-driven models continue to evolve.

Overall, the results advocate for a more personalized and physiologically informed echocardiographic approach, integrating ethnicity, anatomy, and loading conditions into routine clinical practice. Such an approach acknowledges that cardiac function in pregnancy arises from the interaction of body size, geometry, vascular properties, and ethnic background rather than from a single determinant, and therefore necessitates tailored interpretative frameworks rather than universal cutoffs.

### 4.5. Future Directions

The present review highlights substantial ethnic variation in maternal cardiac adaptation during late pregnancy, but several important avenues for future research remain unexplored.

First, because all included studies focused on the third trimester, it is unclear how these ethnic differences emerge, progress, or resolve across the full peripartum trajectory. Longitudinal studies beginning in early pregnancy and extending into the postpartum period are needed to determine whether Asian and Western women follow distinct remodeling pathways, whether the magnitude of ethnic differences widens or narrows over gestation, and whether postpartum regression to baseline occurs at similar rates. Such work would clarify whether the observed third-trimester differences reflect early divergence in myocardial adaptation or represent late-stage consequences of cumulative hemodynamic load.

Second, the “mechanical theory” proposed in this review—emphasizing the interaction between ventricular size, thoracoabdominal geometry, and effective afterload—should be tested in longitudinal designs. Serial assessment of thoracic configuration, diaphragmatic position, ventricular geometry, and central hemodynamics would help determine whether changes in strain within each ethnic group track with predictable mechanical shifts across gestation, and whether ethnic differences represent distinct mechanistic pathways or simply differing positions along the same physiological continuum.

Third, the structural and biomechanical framework explored in this review does not capture the full spectrum of factors that may shape maternal cardiac function. Unmeasured determinants—including nutritional profiles, physical activity patterns, environmental exposures, socioeconomic factors, and genetic polymorphisms influencing calcium handling or sarcomeric proteins—may contribute meaningfully to interethnic variation in myocardial performance. These domains remain almost entirely unexplored in pregnancy research but could interact with geometry and loading to influence systolic function.

To address these gaps, future prospective studies should incorporate detailed assessment of lifestyle and behavioral factors, objective measures of physical activity, standardized nutritional evaluations, and—where feasible—genetic or biomarker analyses reflecting myocardial or vascular phenotype. Optimally designed cohorts would combine standardized echocardiography with central blood pressure or pulse-wave analysis, thoracic biometric measurements (including the modified Haller Index), and harmonized reporting of anthropometric data. Such multidimensional designs will be essential to disentangle the relative contributions of geometry, environment, vascular physiology, and biology to pregnancy-related cardiac adaptation across diverse populations.

### 4.6. Limitations of the Included Studies

Several limitations inherent to the primary studies should be taken into account when interpreting the findings of this review.

First, although all studies enrolled healthy third-trimester pregnant women, methodological rigor was generally modest. None of the investigations provided a formal sample size justification, and blinding of echocardiographic assessors was rarely reported, introducing potential measurement bias.

Second, despite the uniform application of transthoracic echocardiography, there was substantial methodological heterogeneity across studies—including differences in ultrasound equipment, imaging protocols, frame rates, speckle-tracking software, and use of 2D versus 3D STE. This heterogeneity likely contributed to the wide variability observed in strain parameters. Importantly, because of this methodological diversity and the aim of the study, our review was conducted as a systematic review rather than a meta-analysis. As such, our primary synthesis is narrative, relying on study-level medians, ranges, and directional consistency. The inverse-variance weighted pooled means included in the Results serve only as descriptive summary indicators and not as formal meta-analytic estimates. Likewise, the forest plots—presented later in the manuscript—were used solely for visualization of inter-study variability, not for inferential purposes.

Third, in the context of such extreme heterogeneity, the pooled *p*-values should not be interpreted as precise inferential measures. We therefore emphasize that these *p*-values illustrate only the overall direction of differences rather than their statistical certainty, and interpretation should instead focus on the distribution and overlap of study-level values.

Fourth, a critical conceptual limitation relates to the categorization of study populations as “Asian” and “Western.” These terms represent broad, heterogeneous umbrella groups and do not capture the substantial intra-continental diversity in genetics, anthropometry, cardiovascular risk, cultural practices, and pregnancy physiology. Nearly all “Asian” studies in this review were conducted in East Asian populations (China and South Korea); therefore, the findings primarily reflect East Asian physiology and may not generalize to South or Southeast Asian populations, which differ meaningfully in body habitus, metabolic risk, and cardiac structure. Similarly, the “Western” grouping encompassed ethnically diverse populations—including Caucasian, Hispanic, and individuals of African descent—yet most primary studies did not report ethnicity with sufficient granularity to allow disaggregation. This lack of detailed ethnicity-specific reporting precluded formal subgroup or interaction analyses. Where limited data were available (e.g., East Asian vs. mixed Western cohorts), the direction of observed differences appeared qualitatively consistent, but a rigorous sensitivity analysis was not possible. Future research must therefore collect standardized, ethnicity-specific individual-level data and avoid treating continental categories as monolithic biological constructs.

Fifth, incomplete reporting of clinical, anthropometric, or hemodynamic variables in several studies limited the ability to account for potentially relevant covariates such as parity, physical activity, or subtle blood pressure differences. Although all pregnancies were normotensive and non-diabetic, unmeasured confounders—including genetic, dietary, or body composition factors—may have influenced myocardial performance. Moreover, most studies were cross-sectional, reducing insight into temporal changes across gestation and limiting conclusions regarding the persistence or progression of the observed ethnic differences.

In addition to limitations of the primary studies, several well-recognized technical constraints of speckle-tracking echocardiography itself must also be acknowledged. STE is subject to inter-vendor variability, as strain values may differ substantially between ultrasound manufacturers and software packages, even when using the same raw images [45]. Results also depend heavily on the operator’s experience, including image acquisition and region-of-interest adjustment during post-processing [46]. Echocardiographic image quality is another critical determinant of tracking accuracy, with suboptimal endocardial definition reducing reproducibility. Likewise, frame rate selection can influence strain measurements: excessively low frame rates impair temporal resolution, whereas excessively high frame rates may compromise speckle stability [47]. Finally, extrinsic mechanical factors, particularly anterior chest wall deformity, breast tissue configuration, or suboptimal acoustic windows, may further compromise tracking reliability [48]. These technical limitations should be considered when interpreting inter-study differences and when comparing Asian and Western cohorts.

Taken together, these limitations underscore the need for larger, multi-center, harmonized prospective cohorts using standardized imaging protocols. Such efforts will be essential to refine population-specific echocardiographic norms and improve the accuracy of cardiac assessment during pregnancy.

## 5. Conclusions

This systematic review demonstrates a coherent, reproducible pattern of ethnicity-associated variation in maternal cardiac adaptation during late pregnancy. Across heterogeneous studies, healthy Asian pregnant women consistently exhibited smaller ventricular chambers together with higher ejection fraction and more favorable longitudinal myocardial deformation, whereas Western women showed mildly attenuated GLS despite preserved global systolic function. Importantly, these findings reflect physiological population-level phenotypes rather than pathology: the Asian pattern represents an efficient systolic profile associated with smaller cardiac size, while the Western pattern is best understood as a load-related, anatomically driven attenuation of longitudinal strain in the context of larger chambers and higher wall stress.

Because this review is intentionally descriptive and not a formal meta-analysis, and given the extreme heterogeneity of the included studies, all pooled summaries must be interpreted with caution. Our approach relied primarily on narrative synthesis, study-level medians, and visual inspection of forest plots, with pooled means presented only as non-inferential summary descriptors. The persistence of group differences even after BSA-indexing suggests that body size explains much—but not all—of the observed variation, and that additional factors such as thoracoabdominal geometry, vascular characteristics, and population-level anthropometric patterns likely modulate myocardial deformation. At the same time, the absence of direct thoracic measurements (e.g., modified Haller Index) or vascular load indices in the primary studies underscores that the proposed mechanical model is hypothesis-generating, requiring validation in future prospective cohorts.

Collectively, these findings highlight the need for more personalized, anatomy-aware, and population-sensitive interpretative frameworks in maternal echocardiography. While ethnicity-stratified norms may provide an initial pragmatic step, future reference standards should integrate body size, age, and thoracic geometric markers to more accurately capture physiological variation across diverse populations. Such refinements will help avoid misclassification of normal ethnic or anatomical variation as subclinical dysfunction and ultimately support more precise cardiovascular assessment during pregnancy.

## Figures and Tables

**Table 1:** Summary of clinical characteristics and principal findings from the Asian studies [17,18,19,20,21,22,23,24,25,26] included in this review. 2D, two-dimensional; 3D, three-dimensional; 4D, four-dimensional; EP, early-onset preeclampsia; GA, gestational age; GDM, gestational diabetes mellitus; GE, General Electric; GHW, gestational hypertensive women; GLS, global longitudinal strain; LP, late-onset preeclampsia; LV, left ventricular; NS, not specified; STE, speckle tracking echocardiography.

Study Name, Publication Year and Country	Size	Mean Age (yrs)	GA (Weeks)	Study Design	STE Software	Main Findings
Cho K.I., South Korea, 2011 [17]	93	30.2	35.1	Prospective, multicenter	GE, 2D-STE	Higher LV-GLS in healthy pregnancy vs. GHW
Cong J., China, Jan 2015 [18]	68	29.6	37.8	Prospective, monocentric	GE, 3D-STE	Significant reduction in LV-GLS in late healthy pregnancy
Cong J., China, Oct 2015 [19]	40	29	36.4	Prospective, monocentric	GE, 3D-STE	Significant reduction in LV-GLS in both EP and LP
Cong J., China, 2016 [20]	62	28.2	38.1	Prospective, monocentric	GE, 2D-STE	Progressive LV-GLS decrease throughout a normal gestation
Yu L., China, 2018 [21]	30	29	33	Retrospective, monocentric	Siemens, 2D-STE	Higher LV-GLS in healthy pregnancy vs. GHW
Sun F., China, 2021 [22]	87	29.9	30.6	Prospective, monocentric	TomTec, 2D-STE	Higher LV-GLS in healthy pregnancy vs. PE
Liu X., China, 2021 [23]	36	28.4	34.5	Retrospective, monocentric	GE, 2D-STE	Higher LV-GLS in healthy pregnancy vs. GHW
Li W., China, 2022 [24]	62	30.7	28.5	Prospective, monocentric	GE, 2D-STE	Higher LV-GLS in healthy pregnancy vs. GDM
Lin X., China, 2023 [25]	30	27.3	34.8	Prospective, monocentric	GE, 4D-STE	Higher LV-GLS in healthy pregnancy vs. GHW
Wang Y., China, 2024 [26]	70	26	29.1	Retrospective, monocentric	NS, 3D-STE	Higher LV-GLS in healthy pregnancy vs. GHW

**Table 2:** Clinical characteristics and main findings of the studies conducted in Western countries [27,28,29,30,31,32,33,34,35,36] included in the present review. 2D, two-dimensional; 3D, three-dimensional; GA, gestational age; GDM, gestational diabetes mellitus; GE, General Electric; GHW, gestational hypertensive women; GLS, global longitudinal strain; LV, left ventricular; PE, preeclampsia; RV, right ventricular; STE, speckle tracking echocardiography.

Study Name, Publication Year and Country	Size	Mean Age (yrs)	GA (Weeks)	Study Design	STE Software	Main Findings
Shahul S., USA, 2012 [27]	17	29	38	Prospective, monocentric	TomTec, 2D-STE	Higher LV-GLS in healthy pregnancy vs. PE
Estensen M.E., Norway, 2013 [28]	65	32	36	Prospective, monocentric	GE, 2D-STE	Lower LV contractility in healthy pregnancy than at 6 months postpartum
Meera S.J., USA, 2017 [29]	72	29.2	26.8	Retrospective, monocentric	Siemens, 2D-STE	Higher LV-GLS in healthy pregnancy vs. GDM
Buddeberg B.S., United Kingdom, 2020 [30]	40	34.8	39.3	Prospective, monocentric	GE, 2D-STE	Higher LV-GLS in healthy pregnancy vs. GDM
Company Calabuig A.M., Spain, 2021 [31]	246	32.4	35	Prospective, monocentric	Philips, 2D-STE	Higher LV-GLS and RV-GLS in healthy pregnancy vs. GDM
Sonaglioni A., Italy, 2021 [32]	50	32.3	36	Prospective, monocentric	Philips, 2D-STE	Progressive decrease in biventricular mechanics throughout a normal pregnancy
Giorgione V., United Kingdom, 2022 [33]	141	32.9	38.3	Prospective, monocentric	GE, 2D-STE	Higher LV-GLS in healthy pregnancy vs. GHW
Company Calabuig A.M., Spain, 2023 [34]	118	32.6	34.1	Prospective, monocentric	Philips, 2D-STE	Not significant difference in LV-GLS and RV-GLS in healthy pregnancy vs. PE
Özbebek Y.E., Turkey, 2025 [35]	40	28.6	29.5	Prospective, monocentric	Philips, 2D-STE	Higher LV-GLS in healthy pregnancy vs. iron deficiency anemia
Ilić A., Serbia, 2025 [36]	64	29	33	Prospective, monocentric	GE, 3D-STE	Higher LV-GLS in healthy pregnancy vs. GHW

**Table 3:** Comparison of demographic, anthropometric, and hemodynamic parameters collected in the included studies evaluating Asian cohorts versus Western cohorts. Data are reported as study-level summary measures, expressed as medians with their corresponding ranges. BMI, body mass index; BSA, body surface area; COi, cardiac output index; DBP, diastolic blood pressure; GA, gestational age; HR, heart rate; SBP, systolic blood pressure; SVi, stroke volume index.

	Number of Studies for Parameter Assessed (Asian vs. Western Participants)	Asian Studies	Western Studies	*p*-Value
GA (weeks)	10 vs. 10 (578 vs. 853)	33.8 (28.5−38.1)	34.7 (28−39.3)	<0.001
**Demographics and Anthropometrics**				
Mean age (yrs)	10 vs. 10 (578 vs. 853)	28.8 (26−30.7)	31.3 (28.6−34.8)	<0.001
BSA (m^2^)	10 vs. 10 (578 vs. 853)	1.73 (1.71−1.76)	1.81 (1.77−1.82)	<0.001
BMI (Kg/m^2^)	10 vs. 10 (578 vs. 853)	25.8 (23.5−28.8)	28.4 (26.3−31.1)	<0.001
**Hemodynamics**				
SBP (mmHg)	7 vs. 8 (442 vs. 672)	111.5 (106.2−118.2)	110.3 (95.3−119)	<0.001
DBP (mmHg)	7 vs. 8 (442 vs. 672)	70 (65−75.1)	68.1 (59.4−74)	<0.001
HR (bpm)	6 vs. 5 (349 vs. 465)	86.6 (82.4−90.1)	81.9 (75−88)	<0.001
SVi (mL/m^2^)	5 vs. 4 (262 vs. 227)	39.1 (35.4−44.4)	38 (36−41.7)	<0.001
COi (l/min/m^2^)	5 vs. 6 (287 vs. 537)	3.9 (3.6−4.2)	3.3 (2.7−4.2)	<0.001

**Table 4:** Comparative analysis of conventional echocardiographic parameters measured in healthy pregnant women during the third trimester across the two groups of included studies. Data are reported as study-level summary statistics, expressed as medians with their corresponding ranges. IVS, interventricular septum; LA, left atrial; LAVi, left atrial volume index; LVEDD, left ventricular end-diastolic diameter; LVEDV, left ventricular end-diastolic volume; LVEF, left ventricular ejection fraction; LVESD, left ventricular end-systolic diameter; LVESV, left ventricular end-systolic volume; LVMi, left ventricular mass index; NR, not reported; PW, posterior wall; RWT, relative wall thickness; sPAP, systolic pulmonary artery pressure; TAPSE, tricuspid annular plane systolic excursion; TTE, transthoracic echocardiography.

	Number of Studies for Parameter Assessed (Asian vs. Western Participants)	Asian Studies	Western Studies	*p*-Value
**Conventional TTE parameters**				
IVS (mm)	7 vs. 2 (412 vs. 137)	8.3 (6.9−8.9)	8.6 (8.2−9)	<0.001
PW (mm)	6 vs. 2 (382 vs. 137)	8.2 (7.1−8.6)	8.2 (8−8.3)	>0.99
LVEDD (mm)	9 vs. 3 (542 vs. 177)	45.7 (42.1−49.3)	48.4 (47−51)	<0.001
LVESD (mm)	5 vs. 2 (262 vs. 112)	27.7 (22.9−30.5)	30.9 (30.5−31.2)	<0.001
RWT	8 vs. 5 (512 vs. 368)	0.34 (0.29−0.39)	0.34 (0.27−0.40)	>0.99
LVMi (g/m^2^)	6 vs. 8 (412 vs. 796)	72.6 (65.4−86.1)	78.2 (63.9−128)	<0.001
LVEDV (mL)	5 vs. 5 (230 vs. 543)	94.3 (85.4−106)	108.6 (76.1−138)	<0.001
LVESV (mL)	5 vs. 5 (230 vs. 543)	37.4 (30−39.9)	44.3 (25.1−59)	<0.001
LVEF (%)	10 vs. 9 (578 vs. 813)	64 (59.2−68.1)	60.8 (54−66.1)	<0.001
E/A ratio	6 vs. 5 (348 vs. 368)	1.32 (1.14−1.54)	1.38 (1.25−1.58)	<0.001
E/e’ ratio	6 vs. 5 (374 vs. 360)	9.9 (6.5−13.3)	7.2 (6.2−8.9)	<0.001
LA A-P diameter (mm)	3 vs. 1 (153 vs. 50)	32 (27−36.7)	38.5 (38.5−38.5)	/
LAVi (mL/m^2^)	6 vs. 5 (323 vs. 595)	23.6 (14.2−34.9)	27.2 (24−30)	<0.001
TAPSE (mm)	0 vs. 5 (0 vs. 494)	NR	22.9 (19.9−26.1)	/
sPAP (mmHg)	1 vs. 2 (87 vs. 90)	19.4 (19.4−19.4)	21.3 (20−22.6)	**/**

**Table 5:** Comparison of myocardial strain parameters obtained from the included studies evaluating Asian and Western cohorts of healthy pregnant women in the third trimester. Data are reported as study-level summary statistics, expressed as medians with their corresponding ranges. GCS, global circumferential strain; GLS, global longitudinal strain; GRS, global radial strain; LASr, left atrial reservoir strain; LV, left ventricular; NR, not reported; RV, right ventricular; STE, speckle tracking echocardiography.

	Number of Studies for Parameter Assessed (Asian vs. Western Participants)	Asian Studies	Western Studies	*p*-Value
**STE-derived parameters**				
LV-GLS (%)	10 vs. 10 (578 vs. 853)	21.1 (18.3−24.5)	19 (16.5−20.6)	<0.001
LV-GCS (%)	6 vs. 4 (274 vs. 203)	21.7 (15−26)	21.6 (19.3−25.5)	0.772
LV-GRS (%)	6 vs. 4 (274 vs. 203)	35.2 (24−50.2)	33.4 (26−53)	0.078
LASr (%)	3 vs. 3 (179 vs. 414)	38.4 (34.8−42.3)	30.5 (6.4−49.2)	<0.001
RV-GLS (%)	0 vs. 5 (0 vs. 494)	NR	20.3 (16−24.1)	/

## Data Availability

Data extracted from included studies will be publicly available on Zenodo (https://zenodo.org, accessed on 1 December 2025).

## Supplementary Materials

The following supporting information can be downloaded at: https://www.mdpi.com/article/10.3390/jcm14248745/s1. Supplementary Materials S1: PRISMA 2020 checklist; Supplementary Materials S2: The full INPLASY record (https://inplasy.com/inplasy-2025-11-0078// accessed on 25 November 2025); Supplementary Materials S3 and S4: NIH Quality Assessment of Asian [17,18,19,20,21,22,23,24,25,26] and Western Studies [27,28,29,30,31,32,33,34,35,36].
