# Supplementary material for: Ethnic Variation in Left Ventricular Size and Mechanics During Healthy Pregnancy: A Systematic Review of Asian and Western Cohorts
Source: J Clin Med. 2025 Dec 10;14(24):8745. doi: 10.3390/jcm14248745 (PMC12734179; doi:10.3390/jcm14248745)
Supplement: Supplementary file 1 [file jcm-14-08745-s001.zip › Supplementary Materials S2.pdf]

International Platform of Registered Systematic Review and Meta-analysis Protocols

# INPLASY

## Left Ventricular Size and Mechanics in Asian vs Western Healthy Pregnant Women: A Comparative Systematic Review

INPLASY2025110078
doi: 10.37766/inplasy2025.11.0078
Received: 24 November 2025
Published: 25 November 2025

Sonaglioni, A; Margola, G; Nicolosi GL; Baravelli M.

**Corresponding author:**
Andrea Sonaglioni

sonaglioniandrea@gmail.com

**Author Affiliation:**
MultiMedica.

**ADMINISTRATIVE INFORMATION**

**Support -** No funding.

**Review Stage at time of this submission -** Completed but not published.

**Conflicts of interest -** None declared.

**INPLASY registration number:** INPLASY2025110078

**Amendments -** This protocol was registered with the International Platform of Registered Systematic Review and Meta-Analysis Protocols (INPLASY) on 25 November 2025 and was last updated on 25 November 2025.

## INTRODUCTION

**Review question / Objective** The present systematic review was primarily designed to compare traditional and advanced echocardiographic parameters obtained from TTE studies implemented with STE analysis of LV mechanics in Western vs Asian third-trimester healthy pregnant women.

**Rationale** Based on existing literature, we hypothesized that Asian third-trimester healthy pregnant women might have a higher LVEF than Caucasian third-trimester healthy pregnant women, due to the presumed association between higher LVEF and small cardiac size in Asian ethnicity. Similarly, given the close relationship between myocardial deformation and chamber geometry, we anticipated that LV-GLS might also be significantly more pronounced (more negative) in Asian vs European healthy pregnant women.

**Condition being studied** Cardiovascular disease represents the leading cause of maternal mortalitworldwide. Approximately two-thirds of these deaths are potentially preventable, and it is estimated that 21% are related to cardiac/coronary conditions and cardiomyopathy. Pregnancy imposes substantial hemodynamic stress on the cardiovascular system, including marked increases in cardiac output, heart rate, and circulating blood volume, which may unmask or exacerbate latent cardiac dysfunction. A comprehensive assessment of cardiac structure and function in healthy pregnant women is therefore essential to improve our understanding of physiological cardiac remodeling and to identify deviations that characterize maladaptation, as observed in pregnant women with obesity or in pregnancies complicated by gestational hypertension or gestational diabetes mellitus.



Sonaglioni et al. INPLASY protocol 2025110078. doi:10.37766/inplasy2025.11.0078 Downloaded from https://inplasy.com/inplasy-2025-11-0078/

## METHODS

**Search strategy** Two independent reviewers (A.S. and M.B.) carried out a comprehensive literature search in PubMed, Scopus, and Web of Science from database inception through October 2025. The objective was to identify all studies evaluating cardiac morphology and function in healthy third-trimester pregnant women using transthoracic echocardiography, with or without speckle-tracking echocardiography. The search strategy included combinations of the following terms: “pregnancy” or “pregnant women,” combined with “echocardiography,” “transthoracic echocardiography,” “speckle tracking echocardiography,” “strain imaging,” “left ventricular strain,” “global longitudinal strain,” or “LV mechanics,” and filtered using ethnicity descriptors such as “Asian,” “Chinese,” “Japanese,” “Korean,” “Indian,” “South Asian,” “East Asian,” “Caucasian,” “European,” or “Western.” No language or temporal restrictions were applied. In addition to database searching, the reference lists of all included papers and relevant narrative or systematic reviews were examined manually to identify additional eligible studies. All discrepancies between reviewers were resolved through discussion or, when necessary, consultation with a third investigator.

**Participant or population** Asian vs. Western cohorts of healthy pregnant women evaluated at the third trimester of pregnancy.

**Intervention** Clinical evaluation, transthoracic echocardiography implemented with STE analysis of LV mechanics.

**Comparator** Asian vs. Western cohorts of healthy pregnant women evaluated at the third trimester of pregnancy.

**Study designs to be included** Observational Cohort and Cross-Sectional Studies.

**Eligibility criteria** Studies were considered eligible when they examined healthy third-trimester pregnant women aged between 18 and 45 years, clearly identified as Asian or Western (Caucasian/European). Eligible designs included prospective or retrospective observational studies with either cross-sectional or longitudinal structure. To be included, studies had to assess cardiac structure or function using transthoracic echocardiography and provide quantitative measurements of at least one echocardiographic parameter such as LV dimensions, volumes, LVEF, diastolic indices, or speckle-tracking–derived indices including LV-GLS, circumferential strain, radial strain, or strain rate. Studies lacking sufficient information to classify participants into either the Western or Asian group were excluded.
Exclusion criteria included studies enrolling pregnant women younger than 18 or older than 45 years, women not examined during the third trimester, or women whose ethnicity did not match the Asian or Western categories being investigated. Studies involving pregnancies complicated by preexisting hypertension, pregestational diabetes mellitus, chronic pulmonary or renal disease, structural heart disease, cardiomyopathy, congenital heart abnormalities, or fetal abnormalities were excluded, as were pregnancies complicated by gestational hypertension, preeclampsia or gestational diabetes mellitus. Studies were also excluded if participants exhibited hemodynamic instability or if echocardiographic images were judged inadequate. Non-eligible publication types included case reports, small case series, editorials, narrative reviews, conference abstracts, and any study relying exclusively on imaging modalities other than echocardiography.

**Information sources** PubMed, Scopus, and Web of Science.

**Main outcome(s)** To compare the effect of pregnancy on LV mechanics in Asian healthy pregnant women vs, Western healthy pregnant women at the third trimester.
Comparisons of pooled mean values between Asian and Western cohorts were performed using Welch’s t-test, which does not assume equal variances and is appropriate for sample size imbalance and heteroscedasticity between groups. This approach allowed consistent computation of p-values across all parameters for which mean and standard deviation were available. Pooled means were derived through inverse-variance weighting, and between-study heterogeneity was quantified using the $I^2$ statistic.
To visualize differences in key functional parameters, forest plots for LVEF and LV-GLS were generated using Python (matplotlib), displaying study-specific means with 95% confidence intervals stratified by ethnicity. A vertical reference line indicated the pooled Western mean to facilitate direct comparison.

**Quality assessment / Risk of bias analysis** The methodological quality of all included studies was assessed independently by two reviewers (A.S. and G.M.) using the National Institutes of Health (NIH) Quality Assessment Tool for Observational Cohort and Cross-Sectional Studies. Each study

Sonaglioni et al. INPLASY protocol 202511078. doi:10.37766/inplasy2025.11.0078 Downloaded from https://inplasy.com/inplasy-2025-11-0078/

was categorized as having good, fair or poor quality in accordance with the NIH scoring criteria. Inter-rater agreement between the two reviewers was quantified using Cohen's kappa, and any disagreements were settled through discussion and consensus.

**Strategy of data synthesis** Two reviewers (A.S. and M.B.) independently screened all retrieved titles and abstracts and subsequently evaluated the full texts of articles meeting initial eligibility criteria. Data extraction was performed using a standardized form that captured study characteristics (including first author, country, year of publication and study design), population characteristics (such as sample size, maternal age, ethnicity, gestational age at echocardiography, parity, body mass index, body surface area, blood pressure and heart rate), and echocardiographic methodology (including equipment manufacturer, STE software vendor, dimensionality of imaging such as 2D or 3D STE, frame rate, and operator experience). Extracted echocardiographic variables included LV dimensions and volumes, LVEF, LV mass index and diastolic indices, as well as all available strain parameters including LV-GLS, LV global circumferential strain, global radial strain, twist, torsion and strain rate indices. Summary statistics were collected as reported in each study. When numerical results were available only in graphical format, values were extracted using digital caliper software. Differences in extracted data were resolved through consensus.

**Subgroup analysis** N/A.

**Sensitivity analysis** N/A.

**Language restriction** No language restriction.

**Country(ies) involved** Italy.

**Keywords** pregnancy; global longitudinal strain; ethnic differences; cardiac remodeling; maternal cardiovascular adaptation.

**Contributions of each author**
Author 1 - Andrea Sonaglioni - Author 1 drafted the manuscript.
Email: sonaglioniandrea@gmail.com
Author 2 - Giovanna Margola - The author provided Publication Bias assessment.
Email: giovanna.margola@studenti.unimi.it
Author 3 - Gian Luigi Nicolosi - The author revised the original manuscript.
Email: gianluigi.nicolosi@gmail.com
Author 4 - Massimo Baravelli - The author read, provided feedback and approved the final manuscript.
Email: massimo.baravelli@multimedica.it

Sonaglioni et al. INPLASY protocol 2025110078. doi:10.37766/inplasy2025.11.0078 Downloaded from https://inplasy.com/inplasy-2025-11-0078/
