# Supplementary material for: Ethnic Variation in Left Ventricular Size and Mechanics During Healthy Pregnancy: A Systematic Review of Asian and Western Cohorts
Source: J Clin Med. 2025 Dec 10;14(24):8745. doi: 10.3390/jcm14248745 (PMC12734179; doi:10.3390/jcm14248745)
Supplement: Supplementary file 1 [file jcm-14-08745-s001.zip › Supplementary Materials S3.pdf]

Criterion (NIH Case–Control Tool)	Cho K.I. et al.	Cong J. Jan 2015	Cong J. Oct 2015	Cong J. 2016	Li W. et al.	Lin X. et al.	Liu X. et al.	Sun F. et al.	Wang Y. et al.	Yu L. et al.
1. Research question/objective clearly stated and appropriate?	YES	YES	YES	YES	YES	YES	YES	YES	YES	YES
2. Study population clearly specified and defined?	YES	YES	YES	YES	YES	YES	YES	YES	YES	YES
3. Sample size justification provided?	NO	NO	NO	NO	NO	NO	NO	NO	NO	NO
4. Controls drawn from same/similar population & timeframe as cases?	YES	YES	YES	YES	YES	YES	YES	YES	YES	YES
5. Case/control definitions valid, reliable, applied consistently?	YES	YES	YES	YES	YES	YES	YES	YES	YES	YES
6. Cases clearly	YES	YES	YES	YES	YES	YES	YES	YES	YES	YES

defined and differentiated from controls?										
7. If <100% of eligible included, were cases/controls randomly selected?	NR	NR	NR	NR	NR	NR	NR	NO	NR	NO
8. Use of concurrent controls (not historical)?	YES	YES	YES	YES	YES	YES	YES	YES	YES	YES
9. Exposure/risk measured before outcome?	NO	NO	NO	NO	NO	NO	NO	NO	NO	NO
10. Exposure measures valid, reliable, applied consistently?	YES	YES	YES	YES	YES	YES	YES	YES	YES	YES
11. Assessors blinded to case/control status?	YES	NR	NO	NO	YES	NR	NR	NR	NR	YES
12. Confounders measured & adjusted for?	NO	NO	NO	NO	NO	NO	NO	NO	NO	NO

NIH quality score	8	7	7	7	8	7	7	7	7	8
