# Supplementary material for: Ethnic Variation in Left Ventricular Size and Mechanics During Healthy Pregnancy: A Systematic Review of Asian and Western Cohorts
Source: J Clin Med. 2025 Dec 10;14(24):8745. doi: 10.3390/jcm14248745 (PMC12734179; doi:10.3390/jcm14248745)
Supplement: Supplementary file 1 [file jcm-14-08745-s001.zip › Supplementary Materials S4.pdf]

Criterion (NIH Case–Control Tool)	Shahul S. et al.	Estensen M.E. et al.	Meera S.J. et al.	Buddeberg B.S. et al.	Company Calabuig A.M. 2021	Sonaglioni A. et al.	Giorgione V. et al.	Company Calabuig A.M. 2023	Özbebek Y.E. et al.	Ilić A. et al.
1. Research question/objective clearly stated and appropriate?	YES	YES	YES	YES	YES	YES	YES	YES	YES	YES
2. Study population clearly specified and defined?	YES	YES	YES	YES	YES	YES	YES	YES	YES	YES
3. Sample size justification provided?	NO	NO	NO	NO	NO	NO	NO	NO	NO	NO
4. Controls drawn from same/similar population & timeframe as cases?	YES	YES	YES	YES	YES	YES	YES	YES	YES	YES
5. Case/control definitions valid, reliable, applied consistently?	YES	YES	YES	YES	YES	YES	YES	YES	YES	YES
6. Cases clearly defined and	YES	YES	YES	YES	YES	YES	YES	YES	YES	YES

differentiated from controls?										
7. If <100% of eligible included, were cases/controls randomly selected?	NR	NR	NR	NR	NR	NR	NR	NR	NR	NR
8. Use of concurrent controls (not historical)?	YES	YES	YES	YES	YES	YES	YES	YES	YES	YES
9. Exposure/risk measured before outcome?	NO	NO	NO	NO	NO	NO	NO	NO	NO	NO
10. Exposure measures valid, reliable, applied consistently?	YES	YES	YES	YES	YES	YES	YES	YES	YES	YES
11. Assessors blinded to case/control status?	YES	NR	NR	NR	YES	NR	YES	YES	YES	YES
12. Confounders measured & adjusted for?	NO	NO	NO	NO	NO	NO	NO	NO	NO	NO

NIH Quality Score	8	7	7	7	8	7	8	8	8	8
